# Post-Operative Quality of Life after Single-Visit Root Canal Treatment Employing Three Different Instrumentation Techniques—An Institutional Randomized Clinical Trial

**DOI:** 10.3390/jcm12041535

**Published:** 2023-02-15

**Authors:** Vathsalya Shetty, Shalini Yelke, Dian Agustin Wahjuningrum, Alexander Maniangat Luke, Luca Testarelli, Luciano Giardino, Ajinkya M. Pawar

**Affiliations:** 1 Department of Conservative and Endodontics, Nair Hospital Dental College, Mumbai 400008, Maharashtra, India; 2 Department of Conservative Dentistry, Faculty of Dental Medicine, Universitas Airlangga, Surabaya City 60132, East Java, Indonesia; 3 Department of Clinical Science, College of Dentistry, Ajman University, Al-Jurf, Ajman 346, United Arab Emirates; 4 Center of Medical and Bio-allied Health Sciences Research, Ajman University, Al-Jurf, Ajman 346, United Arab Emirates; 5 Department of Oral and Maxillo-Facial Sciences, Sapienza University of Rome, Via Caserta 06, 00161 Rome, Italy; 6 Independent Researcher, 88900 Crotone, Italy

**Keywords:** endodontics, root canal treatment, file systems, rotating files, reciprocating files, post-endodontic pain (PEP), quality of life (QoL)

## Abstract

Root canal treatment (RCT) eliminates damaged pulpal tissue and protects the tooth from recurrent microbial invasion. Post-endodontic pain (PEP) is a frequently encountered complication of root canal therapy. It can have an impact on patients’ quality of life (QoL) and their subjective perception of treatment options. Thus, a self-assessment questionnaire was used to evaluate and compare the influence of manual, rotary, and reciprocating file shaping procedures on immediate post-operative quality of life (POQoL) involving single-visit root canal therapy. It was a double-blinded, randomized, controlled clinical trial. A total of 120 participants were randomly assigned sequentially to three groups comprising 40 patients in each group: Group A: Hand K file (positive control); Group B: ProTaper Next file system; and Group C: WaveOne Gold. Post-operative pain was evaluated using a 4-point visual analog scale (VAS) after 12 h, 24 h, 48 h, 72 h, and 1 week. The highest post-operative pain was with manual instrumentation using hand K files, and the lowest was with reciprocating and rotating instrumentations. No significant difference was noted between the parameters of quality of life assessed, suggesting the filing system or technique had a similar effect.

## 1. Introduction

Root canal therapy (RCT) is a conservative therapeutic option that entails the removal of diseased pulpal tissue and prevents or treats pulpal/periradicular pathosis and safeguards the treated tooth from recurrent microbial invasion [1,2].

Post-endodontic pain (PEP) is a hallmark symptom of root canal therapy and a determinant of long-term failure. PEP typically develops because of acute inflammation in the tissues surrounding the root. Numerous factors can contribute to it, such as preparation that extends past the apical endpoint, insufficient pulp tissue removal, excessive root canal filling material, and the extrusion of dentinal and pulpal debris into the periapical region. Even when the preparation is kept at the apical endpoint, apical extrusion can still happen while using any instrumentation approach, whether manual or mechanical [3]. During canal instrumentation, dentine chips, pulp tissue fragments, necrotic tissue, bacteria, and intracanal irritants may frequently be extruded into the apical foramen, causing post-operative pain.

Debris extrusion has been reported to vary between various rotary systems, which can be attributed to variations in cross-sectional geometry, cutting blade design, taper, flute depth, tip and file sequence, kinematics, and cutting efficacy. Additionally, it has been proposed that the reciprocating motion itself may contribute to the packing of the debris into the irregularities of the root canal space and pushing them past the apical foramen, raising the possibility of post-operative discomfort. Employing nickel–titanium (NiTi) shaping instruments in rotary or reciprocating motion reduces cycle fatigue and improves root canal centering ability. Because varying amounts of irritants are extruded from the root canal area, different nickel–titanium (Ni-Ti) rotational systems may produce varied patterns of neurogenic inflammatory response in the periodontal ligament [4]. The reciprocating WaveOne Gold (WOG) system was introduced, and it has significant alloy, taper, and section enhancements. Additionally, a single point of contact between the instrument and the canal walls is provided by a reduced variable taper and an off-centered, parallelogram cross-section, giving more room for debris clearance. In comparison to conventional mechanical files, the novel characteristics are expected to result in improved conservative shaping, less debris extrusion, and a better post-operative quality of life. Post-endodontic discomfort of mild (10–30%) and severe (6–12%) intensities has been reported in the literature with both manual and rotary file methods, even when the strictest guidelines are followed [5].

Sodium hypochlorite (NaOCl) is the most preferred irrigant administered during root canal therapy because of its many advantages, including its antibacterial activity, antibiofilm activity, and organic tissue dissolving effectiveness. However, it can irritate periapical tissues, particularly at high concentrations, and can elicit an inflammatory response even at concentrations as low as 0.5%, which may indicate a higher chance of post-operative discomfort [6].

Patient quality of life (QoL) and subjective assessments of treatment options can both be impacted by post-operative pain. In the context of a person’s expectations, comfort is a complicated issue relating to socioeconomic, cultural, and health demands. It is an annoying experience for the patient, undermining the patient–clinician relationship. QoL following dental treatment can be evaluated through self-assessing questionnaires such as oral health-related quality of life (OHIP) and post-operative quality of life (POQoL) surveys. The subjective evaluation of the therapy’s effect on the quality of life for seven days after treatment is used in POQoL assessments, which gauge how everyday activities, including eating, speaking, sleeping, and engaging in social interactions, are affected [5]. 

In earlier studies that examined the relationship between root canal therapy and patient quality of life, it was discovered that many patients perceive it negatively because it is frequently associated with pain. More consideration is being given to the viewpoints of the patients in their care, and post-operative QoL may be used to assess the overall efficacy of endodontic therapy [4,5]. 

Few studies have suggested that rotary shaping is related to a higher post-operative quality of life because it reduces debris extrusion past the apex during canal instrumentation [1]. Spohr et al. concluded that more carefully planned research is required before it is possible to make definitive conclusions about the incidence of post-operative discomfort following the use of rotary and reciprocating devices [7]. However, there is no research that considers instruments with the same type of motion but different alloy properties or design decisions. Consequently, using a self-assessment questionnaire, this study was conducted to analyze and compare the effects of manual, rotary, and reciprocating file-shaping approaches on immediate POQoL following single-visit root canal therapy.

## 2. Materials and Methods

This randomized controlled clinical trial (three parallel groups design) was prepared and presented in accordance with CONSORT criteria [8]. This study was authorized by Nair Hospital Dental College (EC/PG-09/CONS/2017 and EC/2020/513) and registered prospectively at Clinical Trials Registry, India (CTRI/2020/03/023880). All patients provided written informed consent to participate in the study, which was carried out in accordance with the principles of the Helsinki Declaration’s most recent edition [9].

### 2.1. Sample Size Calculation

The sample size was calculated using G-Power 3.1.9.2. A power analysis was performed based on where α = 0.05, Power = 0.80, and Effect size 0.91. A total of 120 sample size was calculated considering loss to follow-up, which was divided into (*n* = 40) in each group. 

### 2.2. Eligibility Criteria

Prospective informed and consenting healthy individuals of both genders who registered at Nair Hospital Dental College’s Endodontic Department were included until the requisite study sample size was attained. Asymptomatic subjects >18 years with irreversible pulpitis in mandibular first molars with straight-line access, without any periapical lesions, were included. Each one was planned for primary root canal therapy and had never received emergency endodontic intervention. Patients with sinusitis, periapical abscess, or face cellulitis were excluded from the experiment due to the likelihood of confounding quality of life perception independent of therapy. Patients using analgesics 12 h before and 24 h after treatment were excluded. 

### 2.3. Intervention

**Figure 1:**
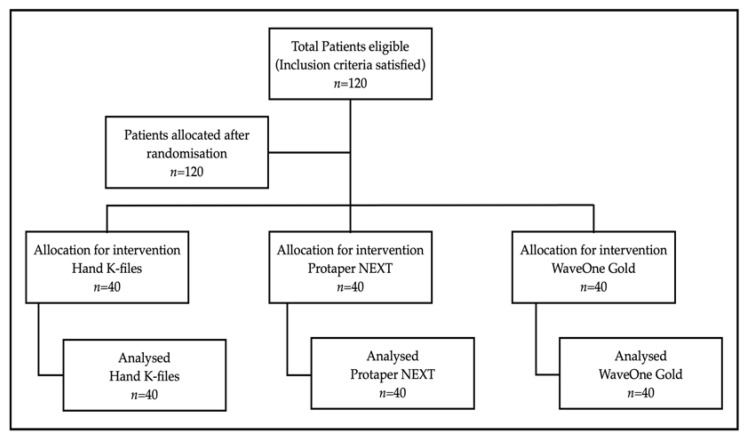
Flow diagram CONSORT for randomized clinical trial.

A complete root canal procedure was performed by a single operator who specialized in endodontics (with experience of more than 10 years) in a single visit to minimize interpersonal variability in the treatment procedures. A thermal test and an electric pulp-testing device (Waldent Innovations Pvt Ltd., New Delhi, India) were used to assess the sensibility of the pulp. The code for different instrumentation techniques was assigned by the principal investigator. The patients were randomly allocated to 3 groups (Figure 1) of 40 each by the dental assistant who did not know the root canal procedure. After evaluating the patient and the tooth that required treatment, the information about the patient and the specific code were written down and sealed in an envelope. Only the operator knew the assigned technique. The participant, the dental assistant, and a second investigator who assessed were unaware of the instrumentation technique. Hence, it was double-blinded. After allocation, the tooth was anesthetized using a cartridge of 2% lignocaine with 1:100,000 adrenaline (Lignox 2%, Indoco Remedies Ltd., Mumbai, India). Each patient was checked for signs of soft tissue anesthesia. Isolation was performed using a rubber dam, and access to the cavity was prepared. Apical patency was confirmed with a size 10 K file (Mani Inc., Tochigi, Japan). The initial working length was determined with an electronic apex locator (Root ZX Mini Apex Locator, Morita Corporation, Kyoto, Japan) and confirmed using radiographs by a blinded operator. Subsequently, root canal procedures were accomplished by any one of the following instrumentation techniques.

### 2.4. Instrumentation Groups 

Group 1: Modified step-back (*n* = 40): The canals were instrumented using a modified step-back technique with stainless steel hand files (Mani Inc., Tochigi, Japan). Minimal preliminary instrumentation was performed using a 15/0.02 hand file. During the modified step-back preparation, coronal 2/3rd was flared using Gates–Glidden burs 2 and 3. Apical 3rd was prepared to the master apical size of 25 K/0.02, and then the step-back technique was performed to 40 K/0.02. The depth of insertion of each larger file was reduced by 1mm. Apical patency was controlled using a 25 K/0.02 file. The flutes of the instrument were cleaned after every instrumentation. A new set of files was used in every study patient. 

Group 2: Rotational technique (*n* = 40): The canal was instrumented using a ProTaper Next (Dentsply Sirona Inc., Charlotte, NC, USA) rotary file. Minimal preliminary instrumentation was performed using a pathfinder file, i.e., Path File, and prepared until X2 (025/0.06) in the sequence of X1 (017/0.04), X2 (025/0.06) at a rotational speed of 300 rpm and 200 g/cm torque according to the manufacturer’s instruction. The instruments were handed down up to the working length. The flutes of the instrument were cleaned after every instrumentation. A new set of files was used in every study patient. 

Group 3: Reciprocating technique (*n* = 40): The canals were instrumented using WaveOne Gold (Dentsply Sirona Inc., Charlotte, NC, USA) files as per the manufacturer’s instruction. Minimal preliminary instrumentation was performed using a Glider file No.015/0.02. The canal was prepared up to a size of 25/0.08 in a slow in-and-out pecking motion combined with a brushing motion. The instrumentation was performed until working length. The flutes of the instrument were cleaned after every instrumentation. A new set of files was used in every study patient. 

### 2.5. Outcomes

After completion of root canal treatment, POQoL and post-operative pain were evaluated. Patients were provided with a questionnaire both before and after their therapy. The question assessed chewing, speaking, sleeping, performing regular chores, social interactions, and general treatment satisfaction.

The patients calibrated and marked their post-operative quality of life affected before the start of the treatment and after the treatment at a definite time interval of 12 h, 24 h, 48 h, 72 h, and at the end of 1 week on an OHIP-14 instrument. The questionnaire was provided in the local language. Each patient received instructions on how to use the questionnaire for the numeric and verbal evaluation of pain discomfort. The post-operative quality of life (POQoL) included response scores on a 5-point Likert scale coded as 0 = Never; 1 = Hardly ever; 2 = Occasionally; 3 = Fairly often; 4 = Very often. 

A trained dental assistant who was blinded to the study procedures instructed the patients to complete a modified Heft–Parker visual analog scale (VAS) to rate their pain at 12 h, 24 h, 48 h, 72 h, and 1-week post-operative intervals. The patients were instructed to take mild analgesics (400 mg of ibuprofen every 6 h) if they felt pain and required pain relief. However, they were also asked to record the number of analgesic tablets on their modified Heft–Parker VAS forms. 

Post-operative pain was evaluated using a 4-point VAS scale after 12 h, 24 h, 48 h, 72 h, and 1 week, where 1: No pain; 2: Mild pain—mild discomfort, no need for treatment; 3: Moderate pain—discomforting, relieved by analgesics; 4: Severe pain—difficult to bear (not relieved by analgesics and unscheduled visit required). 

### 2.6. Randomization 

Computer-generated tables were used to establish the randomized sequence. An operator who was not administering the clinical therapy generated blinded envelopes with each patient’s randomized allocation. The allocation was given to the clinician by the same operator after the initial patient evaluation and before root canal instrumentation.

### 2.7. Data Analysis 

The collected data were statistically analyzed using the Statistical Package for the Social Sciences (SPSS Version 23; Chicago Inc., Northlake, IL, USA). Data were compared using particular statistical tests to determine the statistical significance of the comparisons. To establish the normality of the data for quality of life and pain metrics for the three separate file-treated groups, the Kolmogorov–Smirnov and Shapiro–Wilk tests were used. Both tests revealed no significant differences, confirming that the data were normally distributed. Variables were compared using mean values and standard deviation. One-way Analysis of Variance (ANOVA) was run to find significant differences between the three groups for quality of life before and after treatment. Paired t-test compared the quality of life in the same group. Repeated measures ANOVA was applied to find significant differences in each group at various time intervals of 12 h, 24 h, 48 h, 72 h, and 1 week for pain. A *p*-value less than 0.05 was considered to be statistically significant. 

## 3. Results

The current study was conducted to assess the quality of life (QoL) in endodontic patients treated before and after treatment when treated with three different types of filing systems. QoL remained similar in all groups for all the domains except Physical Disability which was statistically significant at *p* = 0.035, with Group 3 having a greater mean followed by Group 1 and Group 2 (Table 1).

No significant difference was noted between the parameters of quality of life assessed after treatment among the groups, as seen in Table 2, suggesting the filing system or technique had a similar effect.

Table 3 shows the quality of life readings in Group 1, assessed before and after treatment. It can be seen that an improvement was noted in each of the QoL domains, with scores reducing from 1.700 to 0.1250 in Q1, 2.5250 to 0.4250 in Q2, 2.050 to 0.1250 in Q3, 2.4250 to 0.3000 in Q4, 2.3750 to 0.0001 in Q5, 2.1000 to 0.0750 to Q6, and 1.9500 to 0.0250 in Q7. It must be noted that all the questions were assessed from 0 to 4, with 0 scored as never and 4 as very often. The last question was reverse scored, with condition worsened scored as 0 to improved graded as 4. Q8 scores improved from 0.4500 to 1.800, as seen in Table 3. Similar findings were noted in Group 2 and Group 3, as seen in Table 4 and Table 5.

**Figure 2:**
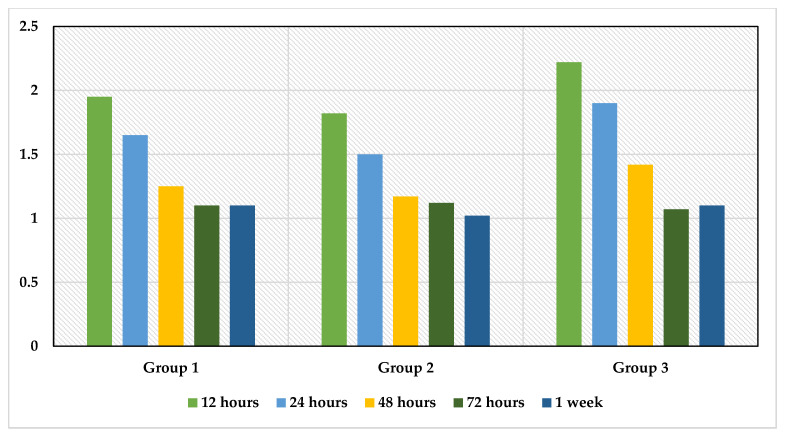
VAS scores across five timepoints for the three experimental groups.

Repeated-measures ANOVA test determined that mean VAS scores differed significantly across 5 timepoints for Group 1 (*F* = 32.603, *p* = 0.000), Group 2 (*F* = 28.530, *p* = 0.000), Group 3 (*F* = 40.734, *p* = 0.000). Therefore, we can conclude that the results for the ANOVA indicate a significant time effect for VAS as measured in all groups, as seen in Table 6, Table 7 and Table 8 and summarised in Figure 2. Between-group comparison for pain readings showed no significant difference across all time intervals, as seen in Table 9.

## 4. Discussion

In order to create safer, more effective, and simpler file systems, manufacturers and researchers have developed modern file systems that combine the best design elements of the past with the most recent technological innovation. The traditional manual K file system and technique was well-liked, but as times changed, newer rotary and reciprocating single-file systems and techniques were added to it [10]. Post-operative pain (PEP) is a common manifestation of root canal therapy, occurring in 1.4–16% of endodontic treatments, and is impacted by the pre-operative condition, treatment approaches employing different file systems, and clinician expertise [3]. PEP could have an impact on the patients’ quality of life (QoL) and their subjective assessment of treatment options [5]. Thus, the purpose of this study was to analyze and compare the influence of manual, rotary, and reciprocating file shaping procedures on immediate POQoL after single-visit root canal therapy using a self-assessment questionnaire. 

In dentistry, QoL may be assessed using two methods: Oral health-related quality of life (OHIP) questionnaires and post-operative quality of life (POQoL) surveys. POQoL evaluation is based on the subjective judgment of the therapy’s influence on QoL for 7 days after treatment and assesses the impairment of everyday activities such as eating, speaking, sleeping, and social connections. The OHIP-14 is sensitive to change in the setting of endodontic therapy and is likely to be effective in assessing patients’ perceptions of endodontic care results. As a result, in this randomized clinical trial, POQoL was assessed using an OHIP-14 questionnaire [1]. 

Pre-operative pain is a predictive factor of post-operative pain. As a result, only teeth with vital pulp that were suggested for endodontic treatment due to various causes were chosen for the current investigation [11]. This study involved mandibular molars. The literature suggests that these teeth are significantly more susceptible to post-obturation pain [12]. Infected debris extrusion is more likely with larger bacterial loads, which can lead to an inflammatory periapical reaction and worse post-operative pain [5]. Researchers that looked into the immunological features of post-operative flare-ups came to the conclusion that antigens coming from the root canal cause the production of an antigen–antibody complex when they are pushed past the apical foramen, which can result in a severe inflammatory response. Substance P and calcitonin gene-related peptide, which activates G protein-coupled receptors on nociceptors and cause the sensitization or activation of neurons, may also be responsible for the pain associated with periradicular inflammation brought on by the extrusion of debris [4,6]. 

The 120 participants were randomly assigned following simple randomization procedures using the lottery method, without any differentiation or discrimination on the basis of age or gender. Patients were sequentially assigned to three groups comprising 40 patients in each group: Group A: Hand K file (positive control); Group B: ProTaper Next file system; Group C: WaveOne Gold. The hand K file (Group A) was used as the control group in the study using the step-back method. Manual technique with stainless steel files for biomechanical preparation has been more popularly used and, thus, was included in our study. In the current clinical trial, ProTaper was chosen to evaluate the impact of a varied taper along the length of the file in contributing to PEP. Based on M-wire technologies, ProTaper Next claims up to 400% reduced cyclic fatigue than the original NiTi instrument. The design features include multiple progressive tapers, which decreases the possibility of taper lock [13]. Reciprocating WaveOne files were chosen for this experiment as a novel intervention to assess the impact of movement kinematics on PEP reduction. Each file in WaveOne Gold features an alternating, offset, parallelogram-shaped cross-section, which is a distinctive design element. The reciprocating system using single-file instrumentation was designed to make the preparation of the root canal faster. This design lessens the taper lock and the screw effect by limiting the engagement between the file and dentin to just one or two sites of contact at any given cross-section [4]. Numerous in vitro studies have shown that the traditional manual step-back technique is associated with more extrusion of the infected remnants or debris as compared to newer rotary techniques [14]. However, in this regard, in vivo studies are few. Therefore, this in vivo study was conducted to evaluate the results of post-operative pain following different root canal preparation techniques using hand K file, rotary (ProTaper Next), and reciprocating systems (WaveOne Gold) and their impact on POQoL following single-visit root canal treatment.

Cold lateral compaction is considered the gold standard against other obturation techniques and, thus, was used in the present study because it has been reported that this technique resulted in minimum post-operative pain comparatively. This was also similar to a study by Shaik et al. in 2022 [3]. Each root canal procedure involved two apical patency procedures to assure the correct detection of the working length and to standardize the clinical protocol. Since it can promote proper cleaning of the apical region of the root canal walls, preventing the development of blocks, ledges, perforations, or apical transportation, this method appears not to enhance post-operative pain [5]. 

There were discernible variations in post-operative discomfort between the three file systems in the current investigation. The duration of pain was the longest in more patients treated with manual instrumentation and least in patients treated with reciprocating instrumentation. Our finding was contrary to that of Atul et al. in 2017, as we found the highest post-operative pain was with manual instrumentation using hand K files, and the lowest was with reciprocating and rotating instrumentations. However, their study findings revealed that K files and ProTaper Next found mild continuous pain in both groups, but the duration of pain was longer for hand instrumentation [11]. Research has suggested that compared to rotary devices, NiTi reciprocating single-file systems may be associated with more severe post-operative discomfort [5,15]. Moreover, a meta-analysis of 42 studies in randomized clinical trials by Hou et al. concluded that the use of reciprocating instruments is associated with a higher incidence of post-endodontic pain than the use of rotary instruments [13]. Randomized clinical trials by Jacoub et al. concluded nonsignificant results when the rotary (PTN) was compared to the reciprocating (WaveOne) system. However, these results were contrary to our investigations [16]. 

When ProTaper rotary and reciprocating WaveOne Gold’s instrumentation were utilized, the pain was minimal. This might be because the flutes on these instruments have a tendency to draw debris back into the orifice, which lowers the amount of extrusion of debris by rotating systems. In contrast, the manual step-back technique tends to plunge the material through the apical foramen, leaving insufficient room for it to be expelled coronally, increasing the likelihood of irritation and pain [4]. Rotating and reciprocating instruments are associated with decreased post-operative discomfort, according to systematic reviews by Hou et al. [13], Sun et al. [17], and Martins et al. [18].

The WOG group had a quicker improvement in the post-operative conditions, which was accompanied by a greater quality of life score in the early posttreatment days. This was likely caused by reduced debris extrusion [19]. Additionally, the flexibility of the new Gold-Wire technology-enhanced instruments may lower the quantity of dentinal debris formed during shaping, resulting in less post-operative discomfort, which would help ease patients’ anxiety [20,21]. 

A study conducted by Shaik et al. in 2022 stated that the incidence of pain followed a similar trajectory, increasing at 8 h in both groups before significantly decreasing at 24 and 48 h [3]. Our finding was in accordance with the findings by Shaik et al. in 2022. We found that at 12 h, higher pain levels were seen, and at 24, 48, and 72 h, the intensity of the pain steadily decreased. Patients treated with the WOG system experienced less discomfort following single-visit endodontic treatment than those treated with the remaining methods. Repeated-measures ANOVA test determined that mean VAS scores differed significantly across five timepoints among the three methods. Another systematic review and meta-analysis conducted by Nobar et al. in 2021 did not find a difference in 12, 24, or 48 h post-operative pain when reciprocating or rotary instrumentation was used for non-surgical root canal treatment [22]. 

In a study conducted by Lakshmi et al. in 2021, “physical pain” and “psychological discomfort” were the post-operative period’s worst effects. Higher scores were also identified in the domains of physical pain and psychological discomfort in studies examining the association between endodontic factors and OHRQoL, demonstrating that symptoms and harm are the first lines of communication for endodontic disorders [6]. In our study, QoL remained similar in all groups for all the domains except Physical Disability which was statistically significant, with WaveOne Gold files having a greater mean followed by hand K files and ProTaper Next files. No significant difference was noted between the parameters of quality of life assessed after treatment amongst the groups suggesting that all the filing systems or techniques had a similar effect. The outcomes of this study and one conducted by Pasqualini et al. are comparable, wherein the quality of life (including pain) of two groups of patients receiving therapy using the rotary approach (ProTaper) and the reciprocating system was compared following treatment (WaveOne) [1]. 

The dentist must provide care that can prevent or treat the side effects of such therapy in addition to conducting root canal preparation with adequate disinfection and pain control. A more holistic approach results in a greater dedication to post-operative care, which unquestionably affects the quality of life. A reduced sample size in each group and the exclusion of variances between single- and multiple-rooted teeth are two limitations of the current study. This observational study’s shortcomings may also be attributed to the patients’ psychological well-being, subjective assessments of their level of discomfort, level of understanding of the questionnaire by the participants, and opinions about root canal therapy. Further prospective studies are needed to ascertain the causative factors of post-operative pain, taking into consideration the central and peripheral pathways of pain. More systematic reviews and meta-analyses need to be documented to help us discover the association of post-operative pain with instrument design and kinematics. 

## 5. Conclusions

Within the parameters and limitations of this randomized clinical trial, the following conclusions can be drawn.

- Hand, rotary, and reciprocating systems tested elicit post-operative pain following single-visit root canal treatment.
- The intensity of post-operative pain varied between the three groups tested. The PTN group exhibited statistically less pain. The order of intensity in the three groups was PTN < hand file < WOG.
- At 12 h, higher pain levels were seen, and at 24, 48, and 72 h, the intensity of the pain steadily decreased.
- No significant difference was noted between the parameters of quality of life assessed, suggesting the filing systems or techniques had a similar effect.

## Figures and Tables

**Table 1:** Comparative evaluation of quality of life before treatment.

Groups	Mean	Std. Deviation	ANOVA Statistic	*p*-Value
**Q1—Functional Limitation**				
Modified Step-back	1.70	1.43	1.182	0.31 (NS)
Rotational Technique	1.75	1.49		
Reciprocating technique	2.17	1.61		
**Q2—Physical Pain**				
Modified Step-back	2.52	1.24	0.155	0.85 (NS)
Rotational Technique	2.60	1.21		
Reciprocating technique	2.45	1.15		
**Q3—Psychological Discomfort**				
Modified Step-back	2.05	1.28	0.410	0.66 (NS)
Rotational Technique	2.30	1.36		
Reciprocating technique	2.27	1.43		
**Q4—Physical Disability**				
Modified Step-back	2.42	1.31	3.436	0.03 * (S)
Rotational Technique	1.95	1.37		
Reciprocating technique	2.70	1.18		
**Q5—Psychological Disability**				
Modified Step-back	2.37	1.39	1.724	0.18 (NS)
Rotational Technique	1.77	1.68		
Reciprocating technique	1.97	1.31		
**Q6—Social Disability**				
Modified Step-back	2.10	1.33	0.148	0.86 (NS)
Rotational Technique	1.95	1.53		
Reciprocating technique	1.95	1.39		
**Q7—Handicap**				
Modified Step-back	1.95	1.61	1.307	2.75 (NS)
Rotational Technique	1.75	1.48		
Reciprocating technique	2.27	1.28		
**Q8—Condition**				
Modified Step-back	0.45	1.17	0.792	0.455 (NS)
Rotational Technique	0.67	1.26		
Reciprocating technique	0.37	0.83		

* = Significant; NS = Not Significant.

**Table 2:** Comparative evaluation of quality of life after treatment.

Groups	Mean	Std. Deviation	ANOVA Statistic	*p*-Value
**Q1—Functional Limitation**				
Modified Step-back	0.12	0.56	0.260	0.771 (NS)
Rotational Technique	0.07	0.26		
Reciprocating technique	0.15	0.53		
**Q2—Physical Pain**				
Modified Step-back	0.42	0.93	1.355	0.262 (NS)
Rotational Technique	0.50	0.98		
Reciprocating technique	0.20	0.56		
**Q3—Psychological Discomfort**				
Modified Step-back	0.12	0.46	1.652	0.196 (NS)
Rotational Technique	0.30	0.75		
Reciprocating technique	0.07	0.47		
**Q4—Physical Disability**				
Modified Step-back	0.31	0.72	0.198	0.821 (NS)
Rotational Technique	0.22	0.53		
Reciprocating technique	0.22	0.57		
**Q5—Psychological Disability**				
Modified Step-back	0.00	0.00	1.187	0.309 (NS)
Rotational Technique	0.02	0.16		
Reciprocating technique	0.07	0.35		
**Q6—Social Disability**				
Modified Step-back	0.07	0.27	0.087	0.916 (NS)
Rotational Technique	0.07	0.27		
Reciprocating technique	0.11	0.38		
**Q7—Handicap**				
Modified Step-back	0.02	0.15	1.707	0.186 (NS)
Rotational Technique	0.12	0.46		
Reciprocating technique	0.25	0.80		
**Q8—Condition**				
Modified Step-back	1.80	1.97	1.495	0.228 (NS)
Rotational Technique	1.77	1.94		
Reciprocating technique	1.15	1.79		

**Table 3:** Comparative evaluation of quality of life before and after treatment in Group 1—Modified Step-back.

Domain/Question	Group	Mean	Std. Deviation	Paired ‘t’ Statistic	*p*-Value
Q1	Before	1.70	1.43	6.565	0.0001 *
	After	0.12	0.56		
Q2	Before	2.52	1.24	8.149	0.0001 *
	After	0.42	0.93		
Q3	Before	2.05	1.28	9.038	0.0001 *
	After	0.12	0.46		
Q4	Before	2.42	1.31	9.604	0.0001 *
	After	0.30	0.72		
Q5	Before	2.37	1.39	10.80	0.0001 *
	After	0.00	0.00		
Q6	Before	2.10	1.33	9.11	0.0001 *
	After	0.07	0.26		
Q7	Before	1.95	1.61	7.65	0.0001 *
	After	0.02	0.15		
Q8	Before	0.46	1.18	−3.406	0.002 *
	After	1.81	1.98		

* = Significant.

**Table 4:** Comparative evaluation of quality of life before and after treatment in Group 2—Rotational Technique.

Domain/Question	Group	Mean	Std. Deviation	Paired ‘t’ Statistic	*p* Value
Q1	Before	1.7500	1.49786	6.725	0.0001 *
	After	0.0750	0.26675		
Q2	Before	2.6000	1.21529	9.416	0.0001 *
	After	0.5000	0.98710		
Q3	Before	2.3000	1.36250	8.832	0.0001 *
	After	0.3000	0.75786		
Q4	Before	1.9500	1.37654	7.817	0.0001 *
	After	0.2250	0.53048		
Q5	Before	1.7750	1.68686	6.488	0.0001 *
	After	0.0250	0.15811		
Q6	Before	1.9500	1.53506	7.623	0.0001 *
	After	0.0750	0.26675		
Q7	Before	1.7500	1.48064	6.946	0.0001 *
	After	0.1250	0.46340		
Q8	Before	0.6750	1.26871	−2.544	0.015 *
	After	1.7750	1.94129		

* = Significant.

**Table 5:** Comparative evaluation of quality of life before and after treatment in Group 3—Reciprocating Technique.

Domain/Question	Group	Mean	Std. Deviation	Paired ‘t’ Statistic	*p* Value
Q1	Before	2.17	1.61543	7.59	0.0001 *
	After	0.15	0.53349		
Q2	Before	2.45	1.15359	10.22	0.0001 *
	After	0.20	0.56387		
Q3	Before	2.27	1.43201	9.13	0.0001 *
	After	0.07	0.47434		
Q4	Before	2.70	1.18105	10.93	0.0001 *
	After	0.22	0.57679		
Q5	Before	1.97	1.31046	8.41	0.0001 *
	After	0.075	0.34991		
Q6	Before	1.95	1.39	7.41	0.0001 *
	After	0.10	0.37		
Q7	Before	2.27	1.28	8.57	0.0001 *
	After	0.25	0.80		
Q8	Before	0.37	0.83	−2.35	0.024 *
	After	1.15	1.79		

* = Significant.

**Table 6:** VAS readings in Group 1 across time intervals.

Time Intervals	N	Mean	Std. Deviation	F Statistic	*p* Value
12 h	40	1.9500	0.74936	32.603	0.0001 *
24 h	40	1.6250	0.66747		
48 h	40	1.2500	0.49355		
72 h	40	1.1000	0.37893		
1 week	40	1.1000	0.37893		

* = Significant.

**Table 7:** VAS readings in Group 2 across time intervals.

Time Intervals	N	Mean	Std. Deviation	F Statistic	*p* Value
12 h	40	1.8250	0.67511	28.530	0.0001 *
24 h	40	1.5000	0.64051		
48 h	40	1.1750	0.38481		
72 h	40	1.1250	0.40430		
1 week	40	1.0500	0.22072		

* = Significant.

**Table 8:** VAS readings in Group 3 across time intervals.

Time Intervals	N	Mean	Std. Deviation	F statistic	*p* value
12 h	40	2.2250	0.86194	40.734	0.0001 *
24 h	40	1.9000	0.95542		
48 h	40	1.4250	0.67511		
72 h	40	1.0750	0.26675		
1 week	40	1.1000	0.30382		

* = Significant.

**Table 9:** VAS readings between groups.

VAS	Mean	Std. Deviation	ANOVA Statistic	*p* Value
**VAS—12 h**				
Modified Step-back	1.9500	0.749	2.855	0.062 (NS)
Rotational Technique	1.8250	0.67511		
Reciprocating technique	2.2250	0.86194		
**VAS—24 h**				
Modified Step-back	1.6250	0.66747	2.841	0.062 (NS)
Rotational Technique	1.5000	0.64051		
Reciprocating technique	1.9000	0.95542		
**VAS—48 h**				
Modified Step-back	1.2500	0.49355	2.331	0.102 (NS)
Rotational Technique	1.1750	0.38481		
Reciprocating technique	1.4250	0.67511		
**VAS—72 h**				
Modified Step-back	1.1000	0.37893	0.198	0.820 (NS)
Rotational Technique	1.1250	0.40430		
Reciprocating technique	1.0750	0.26675		
**VAS—1 week**				
Modified Step-back	1.1000	0.37893	0.351	0.704 (NS)
Rotational Technique	1.0500	0.22072		
Reciprocating technique	1.1000	0.30382		

NS = Not Significant.

## Data Availability

The data presented in this study are available on request from the corresponding author.
